# Editorial: Mesenchymal Stromal Cells: Preclinical and Clinical Challenges

**DOI:** 10.3389/fcell.2022.969178

**Published:** 2022-07-18

**Authors:** Joan Oliva, Simone Pacini, Josep M. Canals, Mayasari Lim

**Affiliations:** ^1^ Department of Clinical Research, Emmaus Life Sciences, Torrance, CA, United States; ^2^ University of Pisa, Pisa, Italy; ^3^ Laboratory of Stem Cells and Regenerative Medicine, Department of Biomedical Sciences, Creatio- Production and Validation Center of Advanced Therapies, Faculty of Medicine and Health Sciences, University of Barcelona, Barcelona, Spain; ^4^ Fujifilm Irvine Scientific, Inc, Santa Ana, CA, United States

**Keywords:** MSC, translational medicine, GMP, regulations, cell/gene therapies

For the past 20 years, mesenchymal stromal cells (MSCs) has become the most studied cell population in the development of adult cell therapies for disease treatment (Keshtkar et al., 2018; Han et al., 2019). MSCs can differentiate into many types of cells (neurons, hepatocytes, myoblast … ) and this is one reason why MSCs hold great promise to the treatment of many immune diseases, cardiac and neurological injuries, and tissue regenerative applications (Hwang et al., 2009). Although MSC research discoveries brought new information, the road for cell therapy approval is still at its dawn. Due to the insurgence of MSC therapies, federal agencies with regulatory oversight to healthcare such as the Food and Drug Administration, European Medical Agency, Pharmaceuticals and Medical Devices Agency, Federal Service for Surveillance in Healthcare, etc. have adapted and continue to update their guidelines as needed (Mendicino et al., 2014; Corbett et al., 2017; Pigeau et al., 2018; Stroncek et al., 2022). Similarly, cell therapy manufacturers and suppliers had to adapt quickly in establishing and adopting best practices that ensure safety, quality, and reproducibility of products and raw materials destined to be used in cell therapy manufacturing. Even so, many questions have surfaced around preclinical tests, scalability of MSC production and clinical application, reproducibility of the results, better characterization of the MSCs, the need for development of defined culture media and GMP compliant animal-free components, ancillary materials, and the development of 3D structures mimicking the tissue organization.

Friedenstein reported the first fibroblastic-like and spindle-shaped cells to differentiate into other type of cells: chondrocyte and osteoblast (Friedenstein et al., 1968; Friedenstein et al., 1974). MSCs have been isolated from different tissues: adipose tissue, dental pulp, periosteum, Wharton’s jelly, umbilical cord (Zuk et al., 2001; Nagamura-Inoue and He, 2014). Due to terminology discrepancies, one of the first task will be to use the exact terminology of MSCs depending on their functional attributes (Bhartiya, 2018). In addition of the Dominici minimal criteria, additional criteria were added for the MSC characterization like MSC responsive to INF-g, TNF-a, indoleamine 2,3 dioxygenase *etc* (Dominici et al., 2006; Bhartiya, 2018), thanks to the formation of international consortium among expert in MSCs. As mentioned by Najar *et al*, Wright et al. and Jayaraman et al., the identification of additional MSC markers will strongly support consistency of data obtained from studies. However, *in vitro,* and *in vivo* results obtained in clinical trials are still not consistent due to lot-to-lot variations, quality of the cells, variability among the donors. To decrease the variability among studies and to increase the reproducibility, standardization of the isolation, identification of additional surface markers, methodology of MSC expansion must be established (Stroncek et al., 2020). A recent study showed that, even if the laboratories use MSCs from the same material source, MSCs showed different behavior in terms of viability after thawing and different transcriptome due to different methodologies used in the lab and donor source (Stroncek et al., 2020). Standardization of MSC isolation, expansion, and characterization, like harmonizing the guidelines for clinicals trials is a major goal for the scientific community. Publications in this Research Topic hopefully will be part of the discussion to help understanding the challenges due to the failure to establish standardized protocols. After the characterization of the MSCs, one of the most important task and challenge encountered is to establish protocols to expand MSCs, that are in harmony and compliance with federal agencies.

There is still a debate around effective delivery and use of MSCs in treating diseases, by injection, by transplantation or by using the secreted extracellular vesicles. The cheapest and fastest methodology is the injection of isolated cells. Injection of cells showed encouraging results, but the long-term effects of MSCs are unknown in terms of treating the diseases, or impairing organ function due to random anchoring of the MSCs. In addition, the survival of injected MSCs is very low (Gyöngyösi et al., 2008), which could explain why treatment of patients is more complicated and not very efficient. Different approaches have been developed to overcome the low efficacy of injected MSCs: increasing the number of injected cells or increasing the number of injections, but it will require a larger scale manufacturing of MSCs, priming the cells, finding the optimal route of administration (Kurtz, 2008; Noronha et al., 2019). The development of cell sheets is an option to control the targeting of the cells in the organs. Myoblast cell sheets were transplanted on heart damaged areas, after heart failure, increasing the period of free events, increasing of survival, and decreasing of death rate. Characterization and the establishment of release criteria before transplantation is a major concern. Actually, only visual observation is used to determine when a cell sheet is ready, and the high variability in outcome is largely due to human dependency based on their experience and knowledge. A more rigorous approach as reported in by Ochiai et al., is to utilize physical characteristics (strength, optical) of the cell sheets to standardize the cell sheets release criteria in GMP facilities.

In addition, the formation of cell sheets modified the production of cytokines by the MSC (Bou-Ghannam et al., 2021). Also, it was noticed that the effect of MSCs in *vivo* studies cannot be explained by the number of cells that reach their target (Bou-Ghannam et al., 2021) indicating that paracrine factors, released by the MSCs, could be the major factor. It is well known that MSCs can modulate the immune system by secreting paracrine factors (Ferreira et al., 2018). MSCs influence inflammation through paracrine factors, which lead to the study of mechanism of action. Many publications, in this research topic, mentioned the importance of EV characterization and variability produced by MSCs, for a beneficial effect on treating different diseases such as spinal cord injury, amyotrophic lateral sclerosis, wound healing, pancreatic cancer, heart failure. As reported by Sykova et al., Johnson et al., Najar et al. and Fernandez-Gonzalez et al, the content of these EV can be altered due to genetic engineering, by priming the MSCs or by using them as a drug transporter, which allow the manipulator to “guide” the EV in a way to have an optimal curative property.

MSCs are used in clinical trials, reported by Garcia-Bernal et al., Sykova et al., Harman and Wiese et al., but as mentioned by Najara, MSCs are used to treat diseases and widely used in clinical trials, but the effect of MSCs on one of the most frequent diseases is still controversial. It is still unclear why MSCs can promote or repress tumors growth/survival. To better understand such opposite effects, retrospective analysis of hundreds of clinical trials is necessary but because reported data are incomplete, the data analysis will be challenging to explain MSC influence on tumors, as mentioned in this research topic by Zhao et al.

Due to the lack of knowledge and experience in MSC clinical applications, federal agencies had to update their guidelines, and keep improving them in parallel to increased pre-clinical and clinical experiences. Food and Drug Administration is a perfect example of how the federal agencies are updating and adding complementary guidelines in the translational field of Cellular and Gene Therapies (Food and Drug Administration, 2022). From 1998 to 2014, FDA released 11 guidelines, but from 2015 to 2022, FDA released 22 guidelines, underlining the importance for agencies to improve their guidelines due to the increase of pre-clinical and clinical studies (Couto et al., 2017; Kabat et al., 2020). However, challenges are still around the corner because many clinical trials published on *clinicaltrials. gov* are still failing to provide detailed information about the patient’s population, the manufacturing of the MSCs (that require a large-scale manufacturing). In addition to the topics mentioned, other crucial subjects about MSCs are presented and discussed in the research topic like priming by Berglund et al., alleviation of ischemia injuries by Arjmand et al. and Chua et al., development of tendinopathy animal model for MSC treatment by Meeremans et al., discovery of new drugs to accelerate MSC differentiation into osteoblast by Wang et al., use of immortalized MSC model for hormonal by Kulebyakin et al., tissue specificity of mesangiogenic progenitors cells by Barachini et al., transcriptional characterization of MSCs by Fan et al. and the impact of MSC senescence state used in clinical trials by Alves-Paiva et al. In order to continue progressing MSC field in clinical applications, academics, clinicians, and industry partners need to continue collaborating, sharing both knowledge and best practices that help to advance this field.
